# Atg8ylation as a host-protective mechanism against *Mycobacterium tuberculosis*

**DOI:** 10.3389/ftubr.2023.1275882

**Published:** 2023-09-29

**Authors:** Vojo Deretic

**Affiliations:** Autophagy, Inflammation, and Metabolism Center of Biochemical Research Excellence, Department of Molecular Genetics and Microbiology, University of New Mexico School of Medicine, Albuquerque, NM, United States

**Keywords:** tuberculosis, autophagy, atg8ylation, macrophage, immunity

## Abstract

Nearly two decades have passed since the first report on autophagy acting as a cell-autonomous defense against *Mycobacterium tuberculosis*. This helped usher a new area of research within the field of host-pathogen interactions and led to the recognition of autophagy as an immunological mechanism. Interest grew in the fundamental mechanisms of antimicrobial autophagy and in the prophylactic and therapeutic potential for tuberculosis. However, puzzling *in vivo* data have begun to emerge in murine models of *M. tuberculosis* infection. The control of infection in mice affirmed the effects of certain autophagy genes, specifically ATG5, but not of other ATGs. Recent studies with a more complete inactivation of ATG genes now show that multiple ATG genes are indeed necessary for protection against *M. tuberculosis*. These particular ATG genes are involved in the process of membrane atg8ylation. Atg8ylation in mammalian cells is a broad response to membrane stress, damage and remodeling of which canonical autophagy is one of the multiple downstream outputs. The current developments clarify the controversies and open new avenues for both fundamental and translational studies.

## Introduction

**Figure 1:**
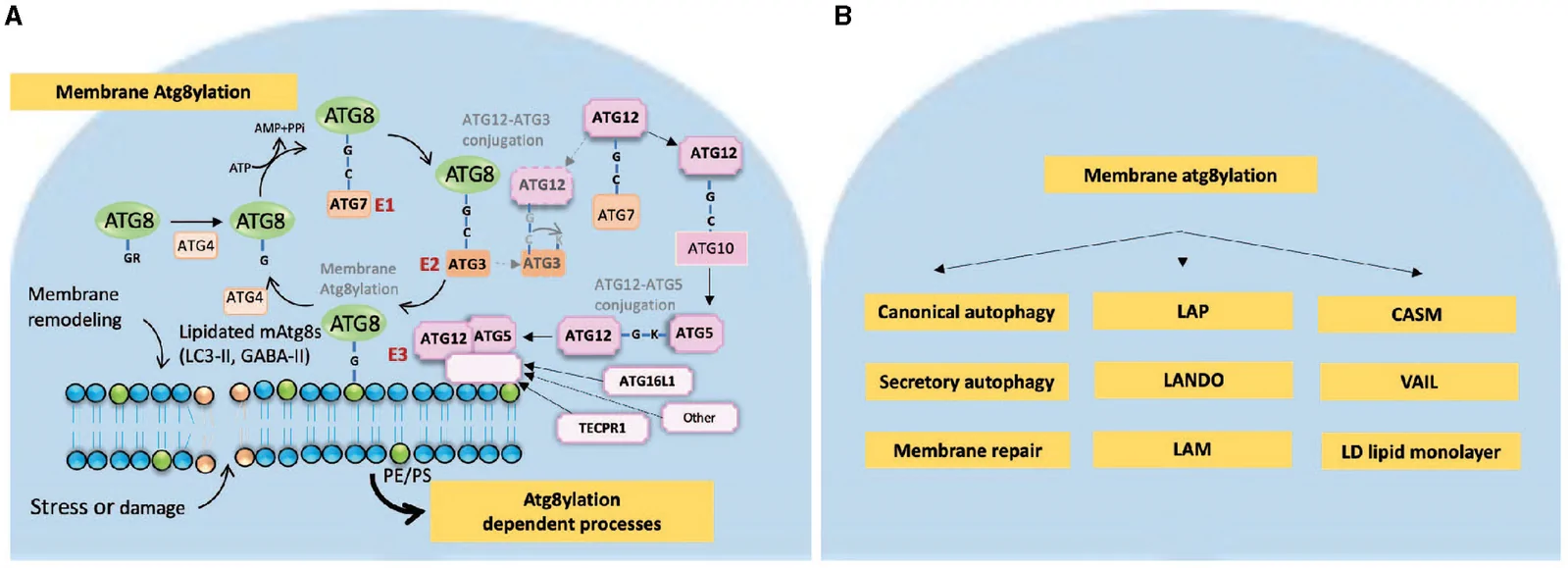
Membrane atg8ylation. **(A)** Membrane atg8ylation process. Membrane atg8ylation is a pathway of covalent membrane modification by ubiquitin-like proteins of the mammalian ATG8 family (LC3A, LC3B, LC3C, GABARAP, GABARAPL1, and GABARAPL2). Membrane atg8ylation occurs in response to membrane stress, damage and remodeling signals and participates in various atg8ylation-dependent processes including canonical autophagy as well as a number of non-canonical and non-autophagy phenomena [listed in **(B)**]. The cycle of membrane atg8ylation and de-atg8ylation resembles the process of protein ubiquitylation and deubiquitylation. It involves activation by ATP of the ATG8 C-terminal Gly residue (once it is exposed following proteolytic processing by ATG4 peptidases) to make a conjugate with E1 enzyme ATG7 via a Gly-Cys high energy thioester bond. ATG8s are then transferred to the E2 enzyme ATG3, which itself has intrinsic affinity for membranes. The final step is the transfer of ATG8s from the ATG8-ATG3 conjugates to form a covalent amide bond with the headgroups of phospholipids such as PE (phosphatidylethanolamine) and alternatively PS (phosphatidylserine). This final step is catalyzed by E3 ligases, which include as a catalytic component the ATG12-ATG5 covalent protein conjugate formed in a separate cycle. ATG12-ATG5 conjugation starts with activation of ATG12, which is another ubiquitin-like molecule and its sequential transfer to ATG7 and ATG10 before making an isopeptide bond between the C-terminal Gly of ATG12 and Lys (K130) of ATG5. The ATG12-ATG5 conjugate binds to a membrane binding component, such as ATG16L1 or TECPR1, to form the E3 holocomplexes (ATG12-ATG5/ATG16L1 or ATG12-ATG5/TECPR1). ATG16L1 or TACPR1 serve to bring to specific membrane sites the ATG12-ATG5 conjugate where it in a spatially restricted manner catalyzes the transfer of ATG8s from the ATG8-ATG3 intermediate to the phospholipids (PE or PS). ATG16L1 recruits the E3 holoenzyme ATG12-ATG5/ATG16L1 to phosphatidylinositol-3-P (PI3P)-marked membrane sites via its binding to WIPI2 protein which in turn recognizes PI3P (omitted from the drawing for clarity). TECPR1 performs identical function by recruiting the E3 holoenzyme ATG12-ATG5/TECPR1 to other membranes tagged by sphingomyelin marks, a situation that can occur on the cytofacial side of endomembranes upon stress or damage. It is postulated here that there are other membrane binding components equivalent to ATG16L1 or TECPR1 that can guide ATG12-ATG5 to other signals on membranes and perform atg8ylation in response to a variety of membrane damage, stress or remodeling cues. The downstream effector processes resulting from atg8ylation of membranes are listed in panel B, whereas the relationship to *M. tuberculosis* host-pathogen interactions including protection of the host are illustrated in the schematic in Figure 2. **(B)** Membrane atg8ylation outputs and downstream processes. Canonical autophagy is a process of formation of autophagosomes in the cytoplasm which can capture and remove by digestion, after their fusion with lysosomal organelles, exogenous or endogenous irritants ranging from microbes or their products to defunct organelles, innate immunity signaling platforms, protein aggregates and danger-associated molecular patterns. At times of starvation, canonical autophagy also plays nutritional role, for example by digesting cytosolic macromolecules and replenishing amino acids and nucleoside pools for biosynthetic needs. Secretory autophagy is an outcome of various canonical and non-canonical autophagy-related processes that lead to exocytosis and release of cargo to the extracellular milieu rather than digestion in lysosomal organelles. Atg8ylation also participates in membrane repair and other adjustments following damage. A wide range of atg8ylation processes on cellular endomembranes (delimiting membranes of various organelles) includes: LAP (LC3-associated phagocytosis), LANDO (LC3-associated endocytosis), LAM (LC3-associated micropinocytosis), CASM (conjugation of ATG8 to single membranes), and VAIL (V-ATPase-ATG16L1-induced LC3 lipidation) (12–14). Of note, atg8ylation requires only the cytofacial monolayer of phospholipids and does not need “single membrane” which by definition consists of a lipid bilayer. This is well-illustrated by atg8ylation of lipid droplets (LD), which are neutral lipid storage organelles delimited by a monolayer of phospholipids that separates the LD core consisting of neutral lipids such as triglycerides and choleteryl esters from the aqueous phase of the cytosol. Note that canonical autophagy, secretory autophagy, LAP, LD stores, V-ATPase, and membrane repair have all been implicated in host-pathogen interactions during *M. tuberculosis* phagocytosis, cytosol invasion, and infection of and replication in macrophages.

**Figure 2:**
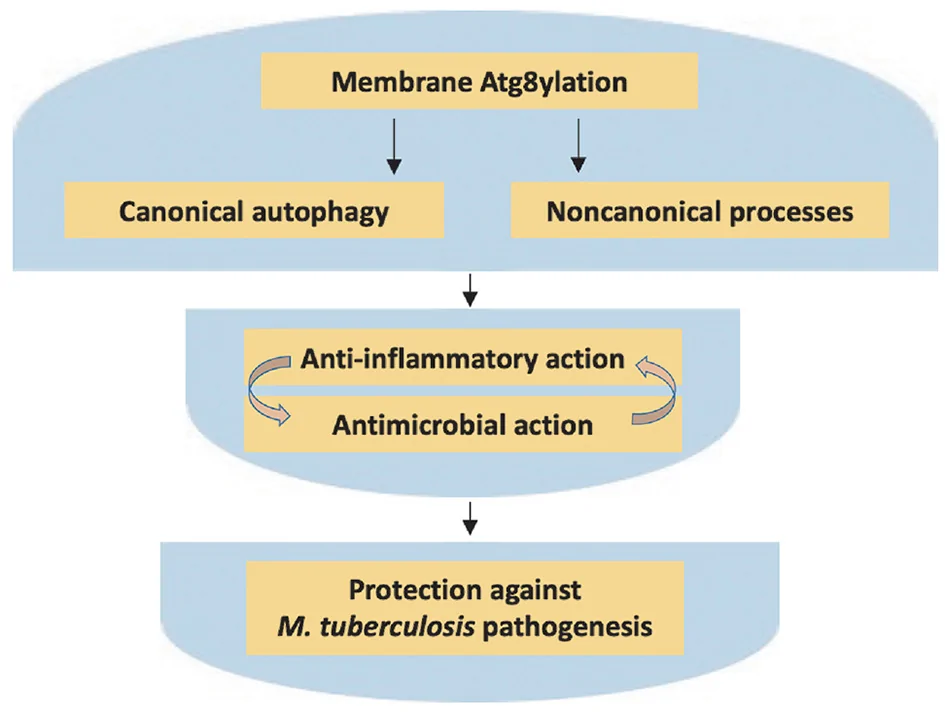
Atg8ylation in control of *M. tuberculosis* pathogenesis. The atg8ylation process and its outputs, as detailed in Figure 1 and this figure, and how it affects host-pathogen interactions in tuberculosis. Both canonical and non-canonical outputs of atg8ylation afford protection against extensive cell-autonomous and tissue inflammation, pro-inflammatory cell death, necrosis, and excessive tissue damage. These processes also contribute to control of intracellular bacteria; note that inflammation and cell death complicate interpretations of the *in vivo* effects on bacterial elimination vs. intracellular bacterial replication. The significance of these processes during tuberculosis infection is supported by the existence of a plethora of *M. tuberculosis* virulence factors that counter atg8ylation outputs, as reviewed elsewhere. All of the above processes, when functional and stimulated by appropriately sequenced immunological responses while avoiding excess or prolonged neutrophilic and other inflammation, contribute to protection of the host reducing tissue damage during *M. tuberculosis* infection.

The most devastating infectious diseases of humans are often the result of novel encounters between the microbe and the human host, as reflected in the recent, past and ongoing pandemics caused by coronaviruses, HIV, infleunza, tuberculosis, and others. They often have history of initial maladaptation between the host and the pathogen causing high lethality, subsequently moderated by the evolution of lineages that balance transmission with pathogenicity. The association between tuberculosis and humans is estimated to had started ca., 50,000–96,000 years ago based on archeological estimates of the most recent common ancestor, with evidence of continuing co-evolution of the pathogen and the host still underway as reflected in different lineages of *Mycobacterium tuberculosis* (*Mtb*) (1) and high mortality of tuberculosis globally (2). The present-day host pathogen interactions in tuberculosis are complex at cell-autonomous and innate and adaptive immunity levels (3–6) underlying the infectious cycle in human populations, including transmission (7), primary active, latent and reactivation disease (8). The clinical presentation and disease outcomes are governed by predisposing conditions, comorbidities, age, coinfections, population and socioeconomic determinants, further complicated by the notorious emergence of antibiotic resistance (8). Among the repertoire of host cell-autonomous defenses and inflammation modulators engaged during *Mtb* infection (6) is the process of autophagy (9, 10), which represents one of the outputs of a broader membrane stress response, termed atg8ylation (11). The topic of this review article is the evolving understanding of membrane atg8ylation (Figure 1A), autophagy and other atg8ylation-dependent processes (12–15) (Figure 1B), with a focus on their anti-inflammatory and antimicrobial effects during *Mtb* infection (Figure 2) and tuberculosis pathogenesis.

## Autophagy and *Mtb* – The early observations

Canonical autophagy (15) has been implicated in defense against *Mtb* (9, 16) contemporaneously with the initial recognition of the broader roles of autophagy in health and disease (17–19) which includes its immune functions (20–24). In principle, autophagy is a homeostatic process that cleanses host cell cytoplasm, turns over long lived proteins and other macromolecules, removes defunct or surplus organelles (15), adjusts cellular metabolic needs (10), and eliminates intracellular microbes and microbial or host products causing inflammation (24, 25). The simplest rendition of autophagy as immune mechanism is that autophagy acts as a cell-autonomous defense (20, 24), whereby it seeks out and eliminates pathogens that manage to penetrate cellular interiors. Autophagy also has a much broader role in innate and adaptive immunity (21, 23, 24). It is of particular significance as a versatile process that suppresses inflammation by removing microbial products collectively referred to as pathogen associated molecular patterns (PAMPs) and endogenous irritants collectively referred to as damage associated molecular patterns (DAMP) or acting upon the signaling platforms that detect PAMPs and DAMPs, the so-called PRRs or pattern recognition receptors (24, 25).

## Autophagy

The mechanism of autophagosome generation in mammalian cells is far more complex than in yeast, the model organism that served to genetically unlock the initial aspects of the canonical autophagy pathway (26). Recently, major strides have been made toward understanding molecular mechanisms driving canonical autophagic membrane formation in mammalian cells via prophagophore and phagophore stages (27–32), phagophore reshaping (33, 34) and expansion via lipid transfer (35–37), cargo capture (38), closure of the nascent phagosomes (39–42), and their fusion with endolysosomal organelles where the cargo is degraded as a primary path to elimination or recycling (43) or alternatively secreted in a process termed secretory autophagy (44).

## Atg8ylation

Membrane atg8ylation, also known as mATG8 lipidation, is the process of covalent modification of various membranes by mammalian ATG8 proteins (mATG8s) that takes place as a response to membrane stress, damage, and remodeling signals (Figure 1A) (11). This process is best understood by analogy to ubiquitylation: atg8ylation is to membranes what ubiquitylation is to proteins (11). The principal protagonists of atg8ylation and ubiqutylation, mATG8s and ubiquitin, are homologous, and the conjugation cascades that activate them and result in membrane atg8ylation or protein ubiquitylation are very similar involving ATP, E1, E2 and E3 ligases (45). The specific factors leading to atg8ylation include two enzymatic cascades with ATG12-ATG5 and mATG8-phosphatidylethanolamine (PE) conjugates as their end products. The former protein-protein conjugate combines with additional proteins to form E3 ligases (46–50) to guide the latter protein-lipid conjugate resulting in atg8ylation of specific membrane domains.

The conjugation cascade follows typical ubiqitylation-like steps (Figure 1A). After proteolytic exposure of the Gly residue at the C-termini, mATG8s are activated by ATP, and transferred to ATG7 (E1) to form mATG8-ATG7 conjugate, then transferred to ATG3 (E2) to form mATG8-ATG3 conjugate and finally to PE in membranes guided by the E3 enzymes. The known E3 enzymes consist of the ATG12-ATG5 conjugate associated with ATG16L1 (46) or (thus far) TECPR1 (47–50). The ATG12-ATG5 conjugate component of the atg8ylation E3 ligases is generated in its own cascade (46). Of note, ATG12 is, like mATG8s, a ubiquitin-like molecule. It is activated by ATP, conjugated to ATG7 (E1), then to ATG10 (E2) and finally to ATG5, forming the ATG12-ATG5 conjugate which then noncovalently associates with ATG16L1 (46) or TECPR1 (48–50) to form E3 ligases directing atg8ylation of different membranes, including autophagosomal and others (11). In preparation for the next step, an ATG12-ATG5 containing E3 ligase activates its substrate mATG8-ATG3 by exposing the thioester bond of the ATG8-G-C-ATG3 intermediate for transfer of the ATG8 to the membrane via formation of an amide bond with the ethanolamine headgroup of the PE phospholipid (46). There are additional branches of these conjugation cascades (Figure 1A), whereby ATG12 can make a non-canonical sidestep conjugate with ATG3 (ATG12-ATG3) (51, 52) which is enhanced in the absence of ATG5 (53).

The specialization of atg8ylation for membranes is ensured by the two extra (relative to ubiquitin) α-helices at the N-terminus of mATG8s with concealed affinities for membranes actuated during atg8ylation (54) and intrinsic membrane affinities of the atg8ylation ligase components ATG3 (55, 56) and ATG16L1 (57, 58). During canonical autophagy, WIPI2, an effector of phosphatidylinositol-3-phosphate (PI3P, a stress-signaling phosphoinositide phospholipid) and a known interactor of ATG16L1 (59–61), helps dock ATG12-ATG5/ATG16L1 and ATG3 to the PI3P-marked membranes to present activated mATG8s for conjugation to the phospholipid PE embedded within the target membrane (58, 62). It is likely that membrane atg8ylation processes other than canonical autophagy may employ yet to be identified functional equivalents of ATG16L1 and WIPI2, as already exemplified with TECPR1 (48–50), to guide atg8ylation on other non-autophagic membranes. Finally, there are exceptions to a strict separation between membrane atg8ylation and protein ubiquitylation as evidenced by crossovers such as atg8ylation of proteins (63–66) and ubiquitylation of membranes (67, 68).

## Manifestations of membrane atg8ylation

There are six principal mATG8s: LC3A, LC3B, LC3C, GABARAP, GABARAPL1 and GABARAPL2 (69–71). The yeast's sole Atg8 has served to delineate the posttranslational modification at the C-terminus of ATG8s with the lipid phosphatidylethanolamine (PE) (72, 73), which can also occur with phosphatidylserine (PS) (74). In mammalian cells, this process, referred to as “LC3 lipidation” and LC3 puncta formation (46, 75), has been widely considered as being synonymous with autophagy and thus has been somewhat indiscriminately used to report observations and measurements of canonical autophagy (76). Whereas LC3B continues to be used to monitor autophagy, it has become evident that it can be present on membranes other than autophagosomes (77). Thus far, mammalian membranes that have been reported to be atg8ylated (covalently modified by mATG8s, typically reported as LC3B but not limited to this mATG8) include: conventional phagosomes harboring pathogens or microbial products (78–81), various types of stressed or signaling endosomal compartments (12–14, 79, 82), lysosomes (48, 66, 83–86), exocytic compartments releasing exosomes (52, 87), ER during its piecemeal ESCRT-dependent lysosomal degradation (88), and lipid droplets (89). Of note, the delimiting membrane of lipid droplets modified by LC3B is not even a full lipid bilayer but a monolayer of phospholipids (89). Thus, the repertoire of cellular membranes that undergo atg8ylation is not limited to double or not even to single membranes. Apparently, all that is needed as a substrate is a PE- or PS-containing phospholipid hemilayer.

Preceding the unified model for atg8ylation (11, 45), the phenomena dependent on atg8ylation have been historically recognized as an assortment of ‘non-canonical autophagy' processes (77) reflected in various terms describing them (Figure 1B): LAP (LC3-associated phagocytosis) (78), LANDO (LC3-associated endocytosis) (90), LC3-associated micropinocytosis (LAM) (82), CASM (conjugation of ATG8 to single membranes) (86, 91), and “vATPase-ATG16L1 axis xenophagy” (80) that later received an acronym VAIL (V-ATPase-ATG16L1-induced LC3 lipidation) (12–14).

Despite a growing plethora of “non-canonical” processes and phenomena that have no autophagic functions and yet engage mATG8s (11, 77), atg8ylation remains an important aspect of canonical autophagy (Figure 2) (11). Although the initial stages of autophagy such as crescent phagophores can form and close in cells lacking all principal mATG8s (42, 65, 92) or in cells defective for mATG8 lipidation (93), their size (92) and contents (93) as well as quality (42) are significantly affected. Among the known functions of the atg8ylation of the autophagosomal membranes are membrane remodeling during autophagosome biogenesis (54, 56, 94–96), its kinetic acceleration (97), the enhancement of selective cargo sequestration into autophagosomes (38), as well as autophagosome-lysosome fusion (98). Recent studies have identified a new role of mATG8s in ESCRT-dependent sealing of autophagic membranes and their maintenance in an impervious state (42). In the absence of mATG8s, the autophagic membrane are permeable to solutes and small macromolecules, arrested at the stage termed amphisome (a hybrid organelles between autophagosomes and multivesicular body endosomes) and cannot progress to autolysosomes (42).

## Atg8ylation, autophagy and *Mtb*

Macrophages are the key cell type where *Mtb* normally replicates and where many aspects of critical host-pathogen interactions occur (3, 99–104). The early seminal studies (9, 16, 105) have shown that induction of autophagy by starvation or IFN-γ, measured by methods available prior to the recent differentiation between atg8ylation and autophagy (11), can restrict virulent *Mtb* in human and mouse macrophages. A number of subsequent studies, carried out either *in vitro* with macrophages and neutrophils or in murine models of tuberculosis, are consistent with the notion that atg8ylation or autophagy (or both) are involved in control of *Mtb* pathogenesis (Figure 2) (53, 106–124). The *in vivo* effects of autophagy on bacterial burden in the mouse models of *Mtb* infection (110, 111) have been reported as negligible (120, 121) or fluctuating (122) depending on the experimental conditions. *In vivo*, any effects directly on bacterial growth (110) are overshadowed by the dominant anti-inflammatory effects of autophagy and atg8ylation as shown in one of the first *in vivo* reports (111) and re-affirmed in subsequent studies (120, 121) (Figure 2). Cell death pathways triggered in infected macrophages (100, 125), modulated by atg8ylation (111, 122, 123), can be proinflammatory or non-inflammatory and may contribute to the inflammation-driven outcomes *in vivo*.

The increased inflammation, persistent neutrophilic infiltration, excessive IL-17 production and Th17 polarization have been initially described in a mouse model of *Mtb* infection with murine *Atg5* inactivated specifically in myeloid cells (*Atg5*^*fl*/*fl*^ *LysM-Cre* mice) (111). A number of studies (126) including those in mice (127) and human populations (128, 129) have shown that neutrophils may contribute to protection early in infection with *Mtb* (130–132) but when they linger for prolonged periods of time, i.e., when Th17 does not mature into Th1 response, they can contribute to tuberculosis pathogenesis (133–138). A study with depletion of neutrophils (120) affirmed the prior findings (111) and additionally demonstrated their key role in causing high mortality of *Mtb*-infected *Atg5*^*fl*/*fl*^ *LysM-Cre* mice (120).

## Revisiting the role of autophagy in control of *Mtb*

The 2004 report that autophagy is a new cell-autonomous defense against *Mtb* (9) was initially validated in 2012 *in vivo* by two groups using mouse models of *Mtb* respiratory infection (110, 111). However, these studies used conditional inactivation of only one Atg gene, i.e., Atg5 (*Atg5*^*fl*/*fl*^ *LysM-Cre* mice). A role for autophagy as a pathway was subsequently challenged in 2015 (120). Whereas, the same report confirmed that *Atg5*^*fl*/*fl*^ *LysM-Cre* mice were more susceptible to *Mtb*, the authors reported that inactivating additional autophagy genes did not increase susceptibility to *Mtb* (120). Worth mentioning is that these were short term studies of up to 80 days post-infection and the efficacy of inactivation of other autophagy genes was not fully validated (120). Nevertheless, this report undercut the burgeoning interest in autophagy as a defense against *Mtb* (139). However, a newer well-designed study demonstrated that dismissal of autophagy was premature and that a more thorough inactivation of additional canonical autophagy genes did confer protection against tuberculosis (122). Shortly afterwards, the same group that initially dismissed autophagy published a revised conclusion (121) that autophagy does matter and that autophagy prevents excessive proinflammatory responses and neutrophil recruitment during *Mtb* infection.

The trajectory of this line of investigation by different groups shows that details do matter and that interpretations of the findings must be scrutinized before making emphatic conclusions. There were both nuanced and major differences in experimental setups of the above studies in murine models of *Mtb* infection deserving further dissection and discussion. The published studies used either low dose (110, 111, 120) or in addition high dose aerosol exposure (111), and the investigators tested chronic (110, 111) or acute (111, 120) disease. Typically, chronic infection initiated by low dose aerosol exposure in mice requires observations of up to 200 days post-infection, dependent on the effect size and penetrance of genetic alterations (53, 110, 122, 140). Acute effects are either observed very early in infection with a low dose (53, 110, 111, 120) or require higher doses of aerosol infection (140–142). The initial analysis (120) of multiple Atg genes with low initial lung deposition of *Mtb* was however limited to short-term, acute disease observations ending at 80 days post-infection. Whereas that study confirmed that *Atg5*^*fl*/*fl*^ *LysM-Cre* mice were particularly susceptible to *Mtb*, i.e., displayed high mortality early on even during low dose infection due to the strength of the effect of Atg5, mice with conditional knockouts in other autophagy genes tested, including Atg14, Ulk1/2 (considered to be the mouse Atg1 orthologs), and LC3-lipidation/atg8ylation enzymatic components Atg3, Atg7, Atg12, and Atg16L1, did not die within the 80 days of reported observation following *Mtb* infection (120). A 2023 study (122) revisited this issue by monitoring mice over a longer, more typical time course, and reported that defects in other autophagy genes, albeit testing only those carrying out atg8ylation (124), rendered mice susceptible to *Mtb*. In this well-executed study, the authors also considered the efficacy of conditional inactivation of the floxed genes (122). They enhanced Cre-driven LoxP-excisions by increasing Cre recombinase gene dosage in mice by breading animals to be homozygous for LysM-Cre (LysM-Cre^+/+^) instead of carrying only one LysM-Cre allele (LysM-Cre^+/−^) used in previous studies (110, 111, 120). This resulted in better inactivation of *Atg16L1* and *Atg7* and in increased susceptibility of Atg16L1^fl/fl^ LysM-Cre^+/+^ and Atg7^fl/fl^ LysM-Cre^+/+^ to *Mtb* compared to their Cre^−^ littermates. Even the mice carrying only one *LysM-Cre* allele (LysM-Cre^+/−^ Atg16L1^fl/fl^) displayed increased mortality when infected with *Mtb*, illustrating the fact that these effects were missed in the previous study limited by shorter duration (120). The Golovkine et al. (122) study reported dynamic changes in bacterial burden in mice with properly Cre-excised floxed *Atg7* and *Atg16L1* genes, characterized by an early lung-specific increase. Regardless of the details on bacterial burden, which fluctuate, all observations agree on increased lesions and neutrophilic infiltration (110, 111, 120) and have been recapitulated in LysM-Cre^+/+^ Atg7^fl/fl^ and LysM-Cre^+/+^ Atg16L1^Fl/Fl^ mice (122).

Collectively, the above studies indicate that multiple autophagy genes are needed for disease control in the mouse model of tuberculosis (Figure 2). They also confirm the well-established notion that *in vivo* autophagy primarily manifests as an anti-inflammatory and tissue-sparing process (21, 24, 25) as reported in one of the two initial studies with *Mtb* infection in mice (111). *Ex vivo*, autophagy can undoubtedly control multiple intracellular microbes in infected cells including *Mtb* and other pathogens (143–149). However, the *in vivo* data with *Mtb* by multiple groups indicate that changes in bacterial burden in infected murine lungs are minor relative to the anti-inflammatory power of autophagy (111), and are not detected at all (121, 150) or fluctuating (122). It is easy to reconcile these apparent discrepancies as fluctuations or minor changes in bacillary loads in the lungs given that autophagy and atg8ylation control inflammation whereas inflammation affects antimicrobial action (Figure 2; middle box) (24), thus confounding bacillary load measurements making data highly dependent on timing and phase of infection. Furthermore as a contributor to modulation of inflammatory responses, in the absence of autophagy (or atg8ylation), cell death occurs more readily thus eliminating cells in which *Mtb* would have an opportunity to replicate. This is further underscored by the findings that absence of autophagy or atg8ylation increases inflammatory responses to microbes (25) and that this paradoxically and artificially protects the host in certain mouse models of infection (151), e.g., with *Listeria monocytogenes* (152), herpesviruses (153), and influenza (154, 155).

Of further import is the fact that the field of autophagy has in the meantime evolved in major ways, and it is necessary to view old and new studies in the broader context of canonical and non-canonical processes. Whereas, these processes are still being linked to the catchall term “autophagy,” they do represent a collection of much wider and disparate membrane stress and remodeling responses (11, 77).

## Separating atg8ylation and autophagy effects on *Mtb*

Mirroring the growth in appreciation of broader biological roles of atg8ylation, of which canonical autophagy is one of the outputs, are the more recent studies with *Mtb*, attempting to differentiate between canonical autophagy and atg8ylation in control of Mtb, both *in vitr*o and *in vivo*. This is a difficult task, as atg8ylation and canonical autophagy are intertwined via the protein conjugation systems (Figure 1A). Furthermore, the full spectrum and ramifications of diverse processes that are autophagy-unrelated or only partially overlapping with autophagy (Figure 1B) are not completely known at this point in time (11, 77).

Interpretation of the available published information is confounded by the emerging relationships and it is difficult to deconvolute atg8ylation-dependent mechanisms capable of controlling *Mtb*. As discussed above, atg8ylation participates in a variety of membrane stress and remodeling responses (11), one of which is LAP (77). Using an *Mtb* mutant in a gene called CpsA (Rv3484) which is susceptible to LAP, an apparent role in control of *Mtb* for LAP has been unmasked (117). This was based on the effects of inactivating/downregulating in murine macrophages genes selectively implicated in LAP (*Rubcn*) or shared with canonical autophagy (atg8ylation factors Atg5, Atg7 and Atg16L1 and Beclin 1), but not in others that are considered specific for canonical autophagy (Ulk1 and Atg14). The susceptibility was observed only during innate immunity responses, as IFN-γ and adaptive immunity abrogated LAP effects both *ex* and *in vivo* (117). Of note IFN-γ induces classical autophagy (9, 105, 115), so it is possible that engagement of atg8ylation machinery in LAP is competing with canonical autophagy for the available atg8ylation resources, as in the case of CASM competing with other atg8ylation processes (86, 156). CpsA inhibits NOX2 (117) implicated in *RUBCN* (Rubicon)-dependent LAP (157). However, complicating the issues, CasA also binds NDP52 and TAX1BP1 (117, 158), two classical receptors involved in selective canonical autophagy of mitochondria and microbes (149). It is also not known how CpsA enters the host cytosol and could this be dependent on permeabilization of phagosomal membranes by ESX-1 (159), a process that induces canonical autophagy (110). In a SCID mouse model of aerosol infection the animals were susceptible to *Mtb* H37Rv but not to its *cpsA* mutant in experiments carried out up to 120 days post-infection (117). It is important to note that while these studies reveal a hidden role for LAP, this role remains masked unless *cpsA* is inactivated and is thus probably not dominant during infection with wild type virulent *Mtb*.

Another recent study addressed the effects of LAP specifically on neutrophilic inflammation in the murine model of tuberculosis (121). Whereas *Atg16L1*^*fl*/*fl*^ *LysM-Cre* mice infected with *Mtb* Erdman showed neutrophilic infiltration akin to the *Atg5*^*fl*/*fl*^ *LysM-Cre* mice, *Rubcn*^−/−^ mice did not. Since *Rubcn* (Rubicon) is key to the atg8ylation process known as LAP (77), based on the above study and its chosen experimental parameters this specific manifestation (LAP) of atg8ylation was deemed not to be involved (121). However, the study (121) did not test *cpsA* mutant, and thus the role of LAP might have remained masked in these experiments. Regardless, the identification of CasA as an inhibitor of LAP and the unmasking of the role of LAP (117) (or perhaps other atg8ylation processes) in *Mtb* pathogenesis represent a major step in deconvoluting different atg8ylation outputs in the context of Mtb infection.

In yet another study with human iPSC-derived macrophages (123), the authors aimed to assess the relative contributions of LAP and canonical autophagy in control of *Mtb*. For this, they (123) compared a strain of *Mtb* (*cpsA* mutant) that cannot inhibit LAP with an *Mtb* strain (*esx* mutant) that is assumed not to elicit canonical autophagy. The latter assumption is based on the notion that *esx* mutant cannot permeabilize the membrane of phagosomes harboring *Mtb*, an event necessary to induce canonical autophagy to counter *Mtb*'s access to the host cell cytosol (110). The loss of ATG7 (which blocks atg8ylation affecting both canonical autophagy and non-canonical outputs including LAP) permitted growth of wild type H37Rv but not of its *esx* and *cpsA* mutants. This affirmed the notion that atg8ylation matters for control of *Mtb* ex vivo but could not discern between canonical autophagy and other non-canonical manifestations of atg8ylation. A loss in iPSC-derived macrophages of ATG14, believed to be specific for canonical autophagy, permitted growth of wild type H37Rv *Mtb* (123), which can be interpreted as an affirmation of canonical autophagy's role in control of *Mtb* in human macrophages (9). Loss of ATG14 also promoted replication of the *esx* mutant (123), contrary to the expectation that *M. tuberculosis* incapable of escaping the phagosome would not be able to induce canonical autophagy (110). This led investigators to propose a further branching of processes controlled by ATGs, whereby ATG14 may affect phagosomal maturation (123) in addition to the conventional role in canonical autophagy.

## Unique effects of atg5 on *Mtb* extend beyond atg8ylation

The mystery of the uniquely penetrant effects of Atg5 on susceptibility to *Mtb in vivo* has recently been partially solved (53). As a reminder, even the latest analyses with a more complete excision of floxed Atg alleles in mice and standard long-term post-infection observations (53, 122) confirmed the uniqueness of the Atg5 phenotype (110, 111, 120). The Atg5^fl/fl^ LysM-Cre^+/+^ mice and even Atg5^fl/fl^ LysM-Cre^+/−^ mice infected with *Mtb* died much faster compared to Atg16L1^fl/fl^ LysM-Cre^+/+^ and Atg7^fl/fl^ LysM-Cre^+/+^ mice (122). In another independent study, Atg5^fl/fl^ LysM-Cre^+/−^ mice succumbed much faster to *Mtb* than Atg7^fl/fl^ LysM-Cre^+/−^ mice (53). Hence, Atg5 has additional roles, past the canonical autophagy and even past the atg8ylation processes (53, 124).

Mechanistically, ATG5 affects exocytosis through multiple mechanisms underlying excessive neutrophil activation during *Mtb* infection (53). In the absence of ATG5, instead of the canonical ATG12-ATG5 conjugate, ATG12 engages in the formation of the non-canonical ATG12-ATG3 conjugate (51) (Figure 1A) which in turn binds the ESCRT protein ALIX (160). This has multiple effects unrelated to autophagy or atg8ylation and is based on ALIX's involvement in maintaining lysosomal membranes intact thus keeping lysosomes in good repair (142, 161, 162). Stress in endolysosomal compartments (66, 140, 142, 159, 163–166) and other membranes (167, 168), which occurs during *Mtb* infection, is exacerbated for lysosomes in ATG5 knockout cells since ALIX is sequestered away by ATG12-ATG3 for the exocytic processes instead of being available for lysosomal repair (53). The pools of ALIX, redirected in the absence of ATG5, augment exocytic events leading to excessive neutrophilic activation and degranulation (53). Thus, the excessive *Mtb* pathogenesis encountered in Atg5^fl/fl^ LysM-Cre^+/−^ mice (111) is caused by hyperactive exocytosis and degranulation by neutrophils further compounded by the inability to maintain functional lysosomes (53).

## Discussion

The focus on canonical autophagy as a potential anti-*Mtb* mechanism has now evolved into a new stage following the improved understanding of the processes controlled by the ATG genes. It has become evident that canonical autophagy is merely a subset of a much broader cellular stress response termed membrane atg8ylation. Atg8ylation pathways that do not engage all parts of the canonical autophagy pathway have many manifestations affecting membranes of various intracellular compartments. This includes variations known under the acronyms LAP, LANDO, LAM, CASM, and VAIL. There is also a ‘sidestep' atypical ATG12-ATG3 conjugation which is enhanced in the absence of ATG5. This atypical ATG conjugate promotes exocytic events, excessive activation of neutrophils and their degranulation, as well as ties up the pools of available ESCRT proteins diverting them away from repair of cellular endomembranes. Several of these processes have already been shown to protect the host against *Mtb* under certain conditions. The improved understanding of atg8ylation and its branching outputs, of which autophagy is one of the best studied but not the only one, offers a new conceptual framework and beckons for cogent repositioning of the field while opening prospects for reformulated fundamental, preclinical, and clinical studies.

## Author contributions

VD: Conceptualization, Writing—original draft.
